# Integration Host Factor of *Mycobacterium tuberculosis*, mIHF, Compacts DNA by a Bending Mechanism

**DOI:** 10.1371/journal.pone.0069985

**Published:** 2013-07-26

**Authors:** Arpit Mishra, Manika Vij, Dhirendra Kumar, Vibha Taneja, Anupam Kumar Mondal, Ankur Bothra, Vivek Rao, Munia Ganguli, Bhupesh Taneja

**Affiliations:** CSIR-Institute of Genomics and Integrative Biology (CSIR-IGIB), Delhi, India; University of Hyderabad, India

## Abstract

The bacterial chromosomal DNA is folded into a compact structure called as ‘nucleoid’ so that the bacterial genome can be accommodated inside the cell. The shape and size of the nucleoid are determined by several factors including DNA supercoiling, macromolecular crowding and nucleoid associated proteins (NAPs). NAPs bind to different sites of the genome in sequence specific or non-sequence specific manner and play an important role in DNA compaction as well as regulation. Until recently, few NAPs have been discovered in mycobacteria owing to poor sequence similarities with other histone-like proteins of eubacteria. Several putative NAPs have now been identified in Mycobacteria on the basis of enriched basic residues or histone-like “PAKK” motifs. Here, we investigate mycobacterial Integration Host Factor (mIHF) for its architectural roles as a NAP using atomic force microscopy and DNA compaction experiments. We demonstrate that mIHF binds DNA in a non-sequence specific manner and compacts it by a DNA bending mechanism. AFM experiments also indicate a dual architectural role for mIHF in DNA compaction as well as relaxation. These results suggest a convergent evolution in the mechanism of *E. coli* and mycobacterial IHF in DNA compaction.

## Introduction

Genome size in various organisms varies from as little as 0.5 Mb in *Mycoplasma* [1] to as much as 3×10^3^ Mb in humans [2]. DNA compaction is hence essential for accommodation in the limited space in the cell. In eukaryotes, DNA is arranged into well defined chromatin structure with the help of histones and high mobility group proteins (HMGs). In bacteria, interplay of DNA supercoiling, macromolecular crowding and association of DNA with histone-like Nucleoid associated proteins (NAPs) enables packaging of the bacterial chromosome into compact structures, called the nucleoid [3]–[5]. Nucleoid associated proteins are rich in basic residues and bind to DNA in sequence specific or non-specific manner. These proteins change the topology of DNA and bring about bending/curving or other architectural changes to compact DNA. Bacterial NAPs play important roles not only in DNA compaction but also in regulation of various cellular processes *viz*., DNA replication, recombination, transcription etc [6].

At least 12 nucleoid associated proteins have been identified in *E. coli* [7]. The major NAPs of *E. coli* that have been studied in details include H-NS (Histone-like nucleoid structuring protein) [8], [9], HU (Histone like protein *E. coli* U93) [10], IHF [11]–[13], Fis (Factor for inversion stimulation) [14], [15], Lrp [16] and Dps (DNA protective protein from starved cells) [17].While H-NS binds DNA non-specifically, it has a preference for intrinsically curved DNA with A/T rich tracts [18]. HU also binds to DNA non-specifically but with a higher affinity for nicked, gapped and cruciform DNA structures [19]. IHF is a sequence homolog of HU, but unlike HU binds consensus DNA motifs in a sequence specific manner with high affinity [20]. Despite differences in sequence specificity or otherwise, bacterial NAPs bring about DNA compaction by one of the three mechanisms of DNA bending, DNA bridging or DNA wrapping. For instance, *E. coli* HU, IHF as well as Fis employ a similar mechanism of DNA compaction by introducing local bends [5]. H-NS, on the other hand, brings about compaction by bridging adjacent tracts of DNA. NAPs, such as Lrp, form oligomers, that enables DNA to wrap around it leading to its compaction [5].

In addition to their architectural roles, several NAPs play important roles in gene regulation as well. For instance, IHF of *Salmonella typhimurium* [11] and *Vibrio cholerae* [21] regulate the protein expression of stationary phase and virulence genes. Fis, on the other hand, acts as a direct activator for many genes involved in translation and flagellar motility [14]. H-NS is a global transcriptional regulator and silences genes involved in virulence and stress response [22], [23] while HU plays an important role in DNA replication, recombination and repair [24].

Nucleoid-associated proteins in mycobacteria, have been discovered only recently, largely due to poor sequence similarities with the canonical and well characterized histone-like proteins and NAPs of *E. coli* and other bacteria. Similar regulatory roles may be hypothesized for the mycobacterial NAPs. However, little is understood about the changes in gene expression brought about by these NAPs during mycobacterial infection or its ability to persist in hostile host environments. Dissecting the interactions and mechanistic details of the unique histone-like DNA-binding proteins of mycobacteria would lead to better understanding of their roles in gene regulation. Of the mycobacterial NAPs identified so far, only HU and Lrp of *M. tuberculosis* share sequence similarity with previously characterized NAPs of *E. coli*. While Lsr2 finds homologs among Actinobacteriaceae, H-NS [25] and GroEL1 appear to be specific to mycobacteria so far [26]. Mycobacterial genomes of *M. smegmatis* and *M. bovis* also encode an integration host factor, designated mIHF, that stimulates the integration of mycobacteriophage L5 [27]. The mycobacterial IHF, mIHF, has several unique properties. It bears no sequence similarity to the *E. coli* IHF and has sequence homologues only among Actinobacteria. Unlike IHF of *E. coli*, mIHF of *M. smegmatis* on its own shows no sequence preference for binding at the attP site. mIHF of both *M. smegmatis* [28] and *M. tuberculosis* [29] are essential for survival of the respective mycobacteria. Recent analysis of mycobacterial proteome puts Rv1388 as the third most abundant protein in mycobacteria [30] highlighting its importance.

IHF of other eubacteria have important roles in gene regulation and one would speculate similar roles for mIHF family. Interestingly, the mIHF homologue, SCO1480 of another Actinobacteria, *Streptomyces coelicolor* has been reported to have an important role in control of sporulation and in antibiotic production [31] [32], suggesting mIHF and its homologues may also act as global transcription regulators. Here, we explore the role of mIHF of *M. tuberculosis*, Rv1388, as a potential nucleoid associated protein of mycobacteria and investigate the architectural role of mycobacterial IHF by atomic force microscopy as well as DNA binding and DNA protection experiments. We demonstrate that mIHF binds DNA in non-sequence specific manner and compacts DNA by a DNA bending mechanism, analogous to *E. coli* IHF family. In addition, AFM experiments indicate a unique dual architectural role for mIHF in DNA compaction as well as relaxation, which depends on the local concentration of the protein. These results suggest that mIHF retains its role in DNA compaction by a similar DNA bending mechanism as the *E. coli* IHF. mIHF, may hence also be implicated as a functional homologue of its *E. coli* counterpart in potential gene regulatory roles.

## Materials and Methods

### Sequence Analysis and Prediction of mIHF ORF

A putative open reading frame of 570 base pairs that may encode a 190 amino acid protein for Rv1388 is annotated as mIHF in TBDB (http://www.tbdb.org) as well as NCBI RefSeq (http://www.ncbi.nlm.nih.gov/RefSeq/). Due to the unusual length of the putative mIHF from *M. tuberculosis* H37Rv when compared to other mycobacterial species, ab initio predictions by Glimmer [33] Genemark [34] and Prodigal [35] algorithms were carried out. In addition, the whole proteome profile of *M. tuberculosis* [36] was analyzed for peptides obtained for Rv1388.

The size of mIHF was confirmed by immunoblotting. Briefly, the cellular extract of late-log phase *M. tuberculosis* H37Rv (generous gift from Tuberculosis Vaccine Testing and Research Materials Contract, Colorado State University) was fractionated on an 18% SDS-PAGE. The proteins were transferred onto nitrocellulose membrane and the blots probed with polyclonal antibody against recombinant mIHF-80 (described below) and developed using SuperSignalR West Pico Chemiluminescent Substrate kit (Pierce Protein Research Products) according to manufacturer’s instructions. A GST-tagged protein from mycobacteria in the lab and purified recombinant mIHF-80 were used as negative and positive controls, respectively.

### Cloning, Expression and Purification of Recombinant mIHF

The open reading frame of Rv1388 (http://www.tbdb.org/) predicts a protein product of 190 amino acids. Gene-specific forward (5′-GCGGATCCATGAGAGACGGAGGA-3′) and reverse (5′-AGGCTCGAGTTAGGCGGAGCCGAAC-3′) primers were used to PCR amplify a 333 base paired fragment of Rv1388 from *M. tuberculosis* H37Rv genomic DNA and cloned at BamHI and XhoI sites in pET28-His_10_-Smt3 vector to give the expression plasmid pmihf-80. This construct yields a protein product lacking the first 79 amino acids of putative full length mIHF and designated mIHF-80. Compared to current annotations in TBDB (http://www.tbdb.org/), pmihf-80 hence consists of a ‘truncated’ mIHF protein comprising residues 80–190 fused to an N-terminal His_10_-Smt3 tag. His-tagged protein was expressed by growing BL21 (DE3) cells transformed with pmihf-80 in presence 50 μg/ml kanamycin. Protein expression was induced by addition of 0.5 mM IPTG, once the cells had grown to A_600_ ≈ 0.5 at 37°C. Cells were further grown with constant shaking at 37°C for 3 hours, harvested by centrifugation and stored at −80°C. All the subsequent steps were performed at 4°C. Cell pellet was thawed on ice and resuspended in buffer A (50 mM Tris-HCl buffer, pH 8.0, 1 M NaCl, 10% (v/v) glycerol). The cells were lysed by sonication, centrifuged to remove insoluble material and the supernatant treated with 5 units of DNase I (Fermentas). The supernatant was then applied to a Ni-NTA agarose column (Qiagen) pre-equilibrated with buffer A. The column was then washed with 20 column volumes of buffer B (50 mM Tris-HCl buffer, pH 8.0, 1 M NaCl, 10% (v/v) glycerol, 10 mM Imidazole) and bound proteins were eluted with 3 column volumes of buffer B containing 250 mM imidazole. The eluted protein was mixed with Smt3-specific protease Ulp1 (Ulp1: protein ratio was 1: 500) and incubated at 4°C overnight to cleave His_10_-Smt3 tag. Tag-free protein was recovered in flow-through by passage of the mixture over fresh Ni-NTA agarose column. Recombinant mIHF-80 thus purified was dialyzed against buffer C (50 mM Tris-HCl buffer, pH 8.0, 500 mM NaCl, 10% (v/v) glycerol). The final salt concentration of the dialysate was adjusted to 150 mM before loading onto an ion-exchange column to remove traces of nucleic acids. Purified protein was pooled, buffer exchanged with buffer C (50 mM Tris-HCl buffer, pH 8.0, 300 mM NaCl, 10% (v/v) glycerol) and concentrated using Amicon ultra 3 kDa molecular cutoff filter units. The concentration of the protein was estimated by Bradford assay (ref) and Bichinconic Acid (Pierce) and stored at −20°C in small aliquots until further use.

### Circular Dichroism Spectroscopy Measurements

Far-UV CD spectra were collected on a Jasco J815 CD spectrometer in a quartz cuvette with a path length of 0.2 cm at room temperature. Ellipticity data were collected in the range of 260–200 nm. CD experiments were carried out with 5 µM mIHF-80 in 12.5 mM Tris-HCl buffer, pH 8.0, 75 mM NaCl and 2.5% glycerol. Each spectrum was recorded as an average of four scans. In all experiments, contributions of the buffer to the spectra were subtracted, and mean residue ellipticities were determined before plotting the spectra.

### Electrophoretic Mobility Shift Assays

Electrophoretic mobility shift assay (EMSA) with DNA was carried out with supercoiled as well as linearized form of pPROEX-HTc (Invitrogen) (linearized with HindIII). Varying concentrations of mIHF-80 ranging from 18 ng to 1500 ng as indicated in figure legends were incubated with DNA in a buffer containing 50 mM Tris-HCl, pH 7.4; 250 mM NaCl; 10% glycerol; 1 mM EDTA for 60 min at 37°C. The protein-nucleic acid complex was analyzed on 0.8% agarose gel by Ethidium Bromide staining.

### DNA Compaction Assay

DNA compaction and condensation upon mIHF binding was confirmed by Ethidium Bromide (EtBr) exclusion assay. A decrease in fluorescence upon mIHF binding of 6.9 kb pET28-derived plasmid DNA intercalated with Ethidium Bromide was monitored and the assay carried out as described previously [37]. Briefly, for each reaction, 4.2 µl of EtBr (15 ng/µl) and 3 µl plasmid DNA (140 ng/µl) such that one EtBr molecule is available per 6 base pairs of DNA were incubated in 96 well plates in dark at room temperature for 5 minutes. 85 µl of mIHF (varying concentrations) was added to each well and further incubated in dark at room temperature for another 60 minutes. EtBr fluorescence was then measured on Infinite M200 Pro multimode reader (Tecan) with excitation and emission at 535 and 595 nm, respectively. The initial fluorescence of DNA with EtBr (no mIHF added) was taken as the maximum, i.e. 100% and the relative percentage decrease in fluorescence signal was calculated and plotted.

### DNA Protection Assay

DNA-protection assay was performed as described previously [26], [38], with slight modifications. 7 µg, 35 µg, 70 µg or 105 µg of mIHF-80 was incubated with 2 µg of a 798 base pair long random linear DNA fragment for 1 h at room temperature in binding buffer (10 mM Tris–HCl pH 7.4, 10 mM NaCl, 0.5 mM EDTA). 1 U of DNase I (Fermentas) was then added and the DNase treatment was carried out for 10 min at 37°C. The reaction was terminated by incubation at 75°C for 10 min, followed by treatment with proteinase K (20 mg) in 5 mM MgCl2, 2% SDS and 0.3 M sodium acetate for 1 hour at 37°C. The protein was separated by phenol: chloroform extraction, the DNA precipitated with ethanol and loaded on a 1% agarose gel in 1X TAE at 120V for 30 min and visualized by EtBr staining.

### Reaction Sample Preparation for AFM

Varying ratios of mIHF-80 with respect to DNA, ranging from 1∶1 to 1∶8 and as indicated in figure legends was incubated with 1 µl of pPROEX-HTc (10 ng/µl), (Invitrogen) at 25°C for 30 minutes. The reaction mix was mixed with 2 µl MgCl_2_ and loaded onto a cleaved mica sheet. The protein-DNA mix was allowed to spread spontaneously and incubated at 25°C for one min to allow molecules to adhere on mica surface. The unbound material was washed with milli Q water and the bound surface allowed to air dry.

### AFM Imaging

Imaging was carried out with 5500 scanning probe microscope (Agilent Technologies, Inc.) using PicoView software. Images were obtained in tapping mode in air with 225-μm-long silicon cantilevers (Agilent Technologies) that have a resonance frequency of around 75 kHz and a force constant of 2.8 Newton/m. Scan speed used was 1 line/s. Minimum image processing (first order flattening and brightness contrast) was employed. Image analysis was performed using PicoImage software v1.4.4. Contour lengths were measured by tracing the molecular contour with a segmented line. End-to-end length was measured from the shortest straight line that connected the ends of an image. All heights were measured in volts as the difference between the top of the biomolecules and the average height of the underlying mica using PicoImage v1.4.4. The image widths were measured from sample/substrate contact points on each side of the biomolecule. Bending angles were determined by evaluating the angle between the centre of the protein and a point 15 nm upstream and downstream on the DNA, as described earlier [39].

## Results

### Comparative Genomics Analysis for ORF of mIHF

**Figure 1:**
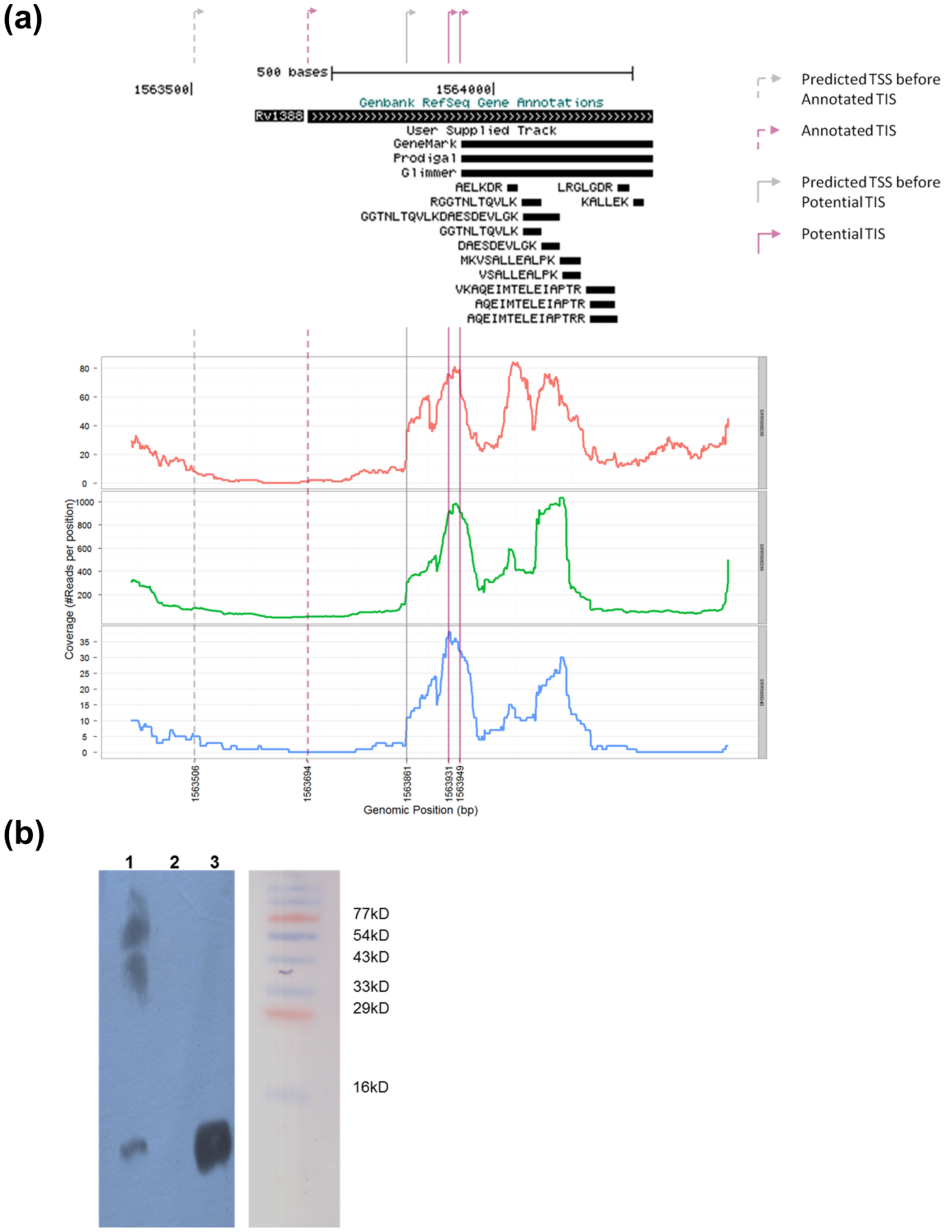
Search for mIHF ORF in M. tuberculosis H37Rv genome. (a)View of genomic locus of Rv1388 with gene predictions, peptide identifications and RNA transcript analysis: Genome annotation of Rv1388 in Genbank (top bar) versus Gene predictions by the indicated algorithms (GeneMark, Prodigal and Glimmer) were visualized in UCSC genome browser. The peptides from proteome analysis [36] are indicated here. Additional whole proteome analyses [40], [41] also indicated peptides corresponding to shorter lengths of Rv1388 and are shown separately for clarity (Fig. S3 in File S1). Details of the identified peptides corresponding to Rv1388 are given in Table S1 in File S2. RNA transcript analysis using two exponential phase data (SRR568038: red and SRR568039: green) and stationary phase data (SRR568040: blue) [42] shows short transcripts which start downstream of the annotated translation initiation site. Read abundance (coverage) at the predicted TSS just upstream of the proposed TIS suggest 111- or 105- residue to be the most likely product. (b)Western blot analysis for mIHF in total cellular extract of M. tuberculosis H37Rv: A western blot using cellular extract of *M. tuberculosis* H37Rv confirms the *in vivo* size of mIHF to nearly 12 kDa (lane 1). Negative controls using GST-tagged mycobacterial protein shows no bands (lane 2), while purified recombinant mIHF-80 (lane 3) is used as a positive control. A pre-stained molecular weight marker of the same gel (before immunoblotting) is indicated.

A search of *M. tuberculosis* H37Rv genome databases for mycobacterial Integration Host Factor suggests an ORF, Rv1388, at the genomic position of 1563694 to 1564263 in the mycobacterial genome (Figure 1a). A BLAST search of Rv1388 against non-redundant protein sequences, reveals Rv1388 belongs to a conserved family of proteins limited to Actinobacteria and closely related organisms but absent in other bacterial groups (Fig. S1 in File S1). *rv1388* encoding for mIHF of *M. tuberculosis* H37Rv and its homologs from *M. tuberculosis* H37Ra, *M. tuberculosis* F11, *M. tuberculosis* KZN or *bcg_1449* of *M. bovis* BCG appear to be unique in this family as the annotations suggest these to encode a 190 amino acid protein (Fig. S2 in File S1). Annotations of other virulent *M. tuberculosis* strains (viz., *M. tuberculosis* Beijing, *M. tuberculosis* Erdman, *M. tuberculosis* Haarlem), or their sequence homologs, on the other hand suggest mIHF of a smaller size of 105 residues. mIHF of the non-pathogenic *M. smegmatis* has been earlier shown to be 105 residues long [27]. In addition, *M. tuberculosis* CDC1551 and *M. marinum* M mIHF homologs are predicted to be of length 111 residues (Fig. S2 in File S1). We investigated whether this apparent discrepancy in the length of mIHF is due to possible misannotations through four different approaches: (i) Firstly, ab initio predictions were carried out using the different algorithms of Glimmer [33], Genemark [34] and Prodigal [35]. All these algorithms suggest *mIHF* gene locus in *M. tuberculosis* H37Rv from 1563949 to 1564263 genomic positions and encoding a smaller product of 105 residue protein (Fig. 1a). (ii) Secondly, multiple whole proteome profile studies of *M. tuberculosis* H37Rv [36], [40], [41] were analyzed for identified peptides of Rv1388. All the peptides identified in these studies belong to the C-terminal fragment of annotated Rv1388 only (Fig. 1a, Fig. S3 in File S1), suggesting that the endogenous protein from this locus may be of a shorter length. However, the absence of a peptide from the putative N-terminus of the 190-residue Rv1388 does not necessarily validate that this region is not part of mIHF *in vivo*. The size of mIHF of *M. tuberculosis* H37Rv *in vivo* was hence further explored using additional approaches. (iii) RNA-seq data of *M. tuberculosis* H37Rv from exponential and stationary phases [42] was analyzed for RNA transcript of mIHF (Fig. 1a). RNA-seq reads mapped onto the *M. tuberculosis* H37Rv genome using the Burrows-Wheeler Aligner (BWA) [43] revealed only a short RNA transcript capable of transcribing a final product of 111 or 105 amino acids under all the conditions investigated. (iv) Lastly, cellular extracts of *M. tuberculosis* H37Rv were analyzed to probe the size of mIHF *in vivo*. Although, endogenous mIHF could not be extracted in large amounts for N-terminal sequencing, the size of mIHF in culture filtrates was found to be nearly 12 kDa by immunoblotting (Fig. 1b), confirming that shorter products are expressed *in vivo*.

Isoforms of a few proteins of *M. tuberculosis* H37Rv have been reported under different conditions. For instance, *sigE* of *M. tuberculosis* H37Rv has been shown to produce an isoform under oxidative but not surface stress [44]. It is likely that the full-length 190-residue mIHF isoform could not be observed under the analyzed conditions. Under exponential and stationary phases analyzed, only a shorter product was identified and appears to be the predominant isoform. Since the size of shorter mIHF isoform could not be unambiguously determined, we chose to use a construct lacking the first 79 amino acids and corresponding to 111 residues (designated mIHF-80) through the course of this work.

### Protein Purification and Features of Recombinant mIHF-80

**Figure 2:**
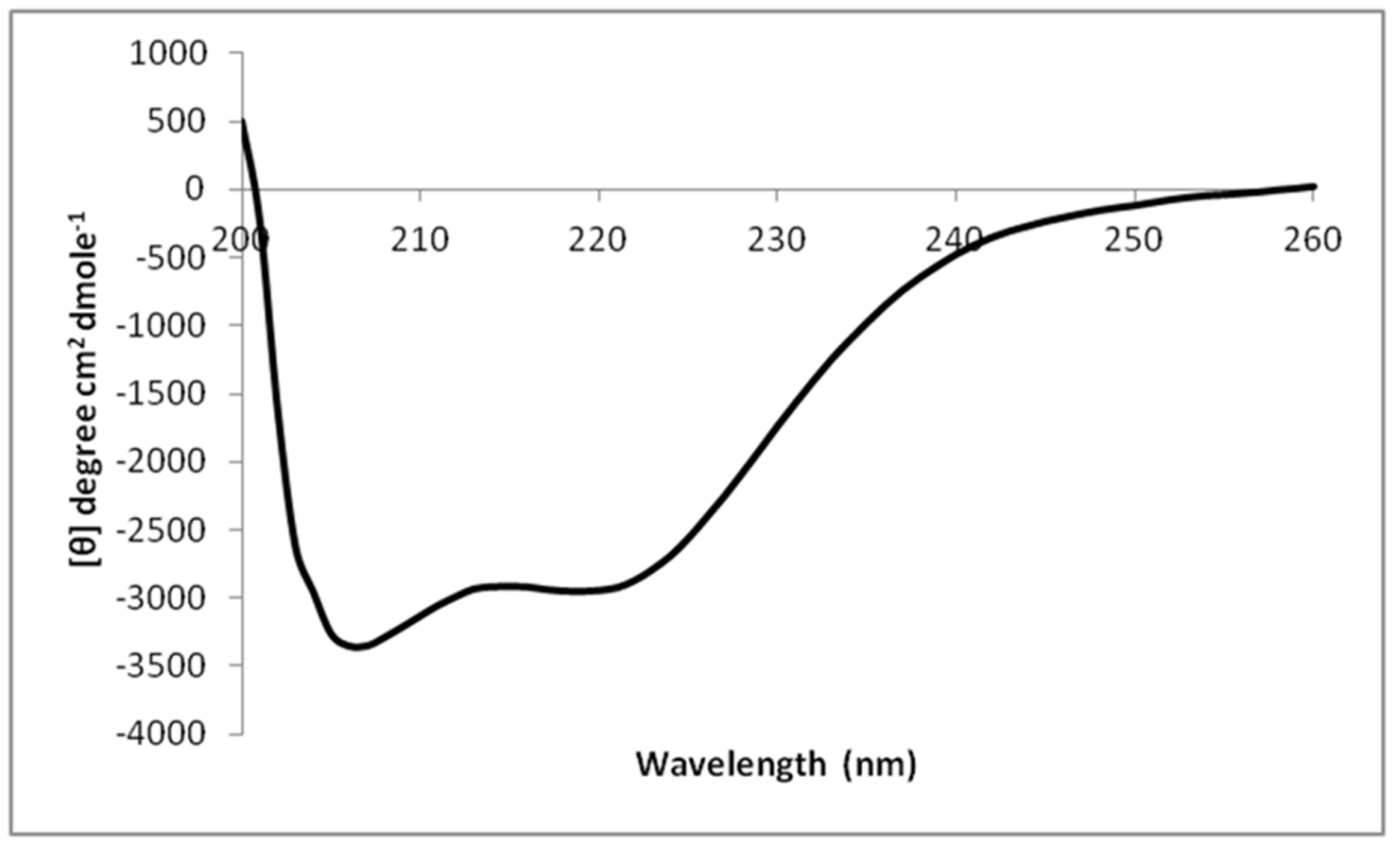
CD profile of mIHF-80. A CD analysis of mIHF-80 is suggestive of a globular, folded protein. The protein appears to be primarily alpha-helical and the helical content calculated to be more than 85% of the total secondary structure content of the protein.

Recombinant mIHF-80 expressed well and was purified from soluble extracts as His_10_-Smt3 fusion at the N terminus over nickel-nitrilotriacetic acid followed by cleavage of the tag by Ulp1 protease. The tag was removed as described in the Methods section and the protein purified to homogeneity (Fig. S4 in File S1). The cleavage site leaves one additional residue (Serine) at the N-terminus of the 111 residues of mIHF-80. Recombinant mIHF-80 exists as a homodimer in solution (Fig. S5 in File S1). The protein is rich in basic residues with more than 20 per cent of its amino acid content comprising of lysines and arginines. Analysis of secondary structure suggests that the protein is rich in helical content (Fig. 2). Both these features are reminiscent of DNA binding proteins.

### mIHF-80 is Involved in Non-specific DNA Binding and Compaction

**Figure 3:**
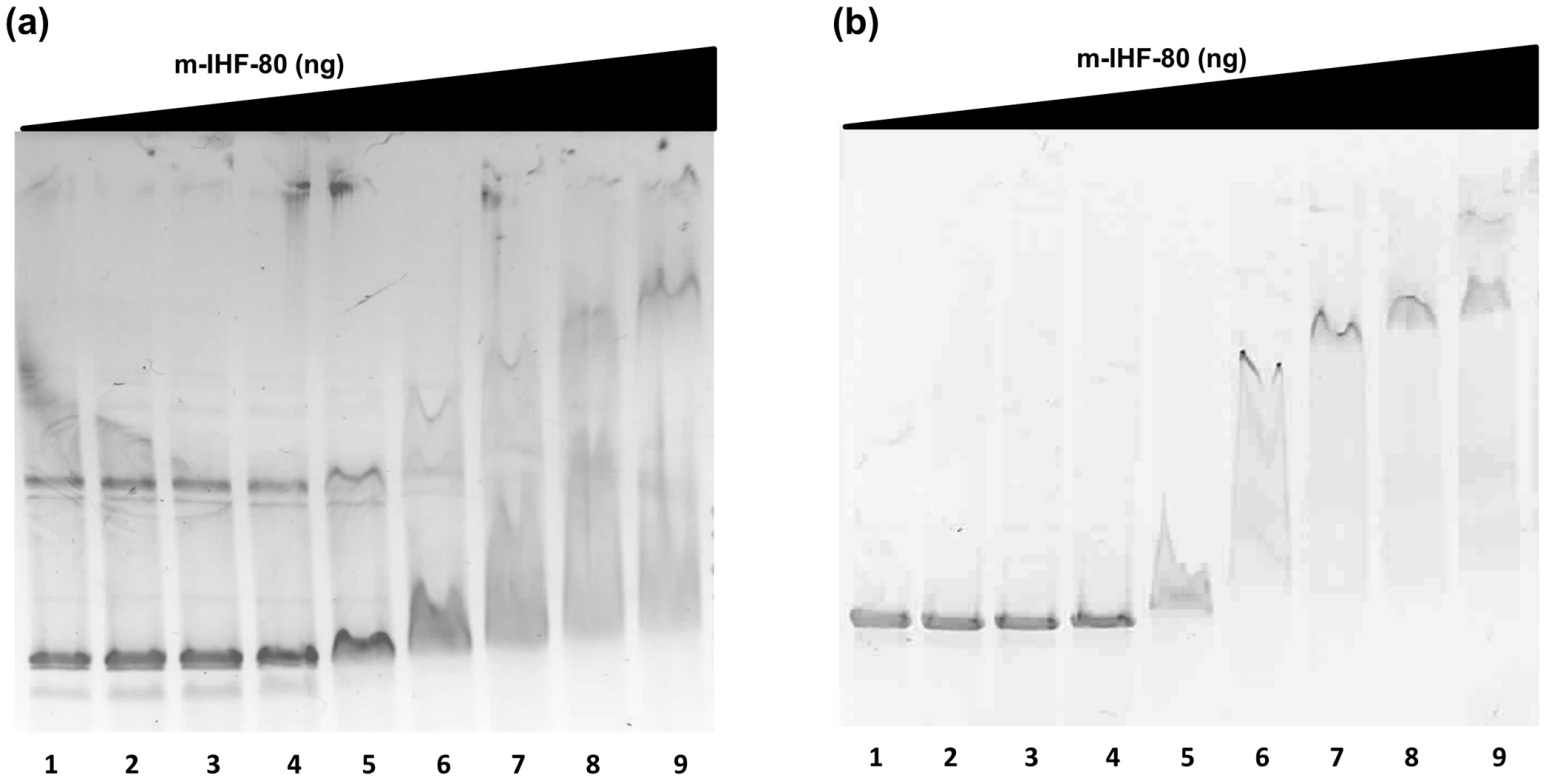
EMSA for nucleic acid binding. Binding of mIHF-80 to (a) circular and (b) linear DNA was analyzed by running the reaction mixture on agarose gel and visualizing by EtBr staining. mIHF-80 binds DNA non-specifically in a concentration-dependent manner. (a) In the reaction mix, 18, 36, 75, 150, 300, 450, 600 or 900, (lanes 2–9) were incubated with circular supercoiled DNA at 37°C for 60 min. (b) To monitor binding to linear DNA, a similar reaction was carried out with 18, 37, 75, 150, 300, 450, 600 or 900 ng mIHF-80 (lanes 2–9) to monitor gel shift. Lane 1 is control with no protein in both gels.

mIHF of *M. smegmatis* has been shown to bind both attP and non-attP containing oligos with similar efficiency [27], [45]. The non-specific DNA binding ability of mIHF-80 was confirmed by EMSA with both linear and circular plasmid DNA. As seen in Fig. 3, mIHF-80 binds strongly to both linear and circular DNA to form stable DNA-protein complexes. An increasing number of complexes (larger levels of gel retardation in Fig. 3) are visible with an increasing protein concentration for both supercoiled as well as linear DNA, confirming that mIHF-80 binds to DNA at multiple sites in a relatively sequence-independent manner. Mycobacterial NAPs, Lsr2 [46], HupB [47] and HNS [48] also bind DNA non-specifically, although they tend to display a preference for A+T rich DNA. Another mycobacterial NAP, GroEL1 does not exhibit this property [26]. The EMSA experiments in the current study confirm mIHF-80 binds DNA in a sequence-independent manner but do not investigate a possible A+T or G+C preference in binding regions and it needs to be further explored.

**Figure 4:**
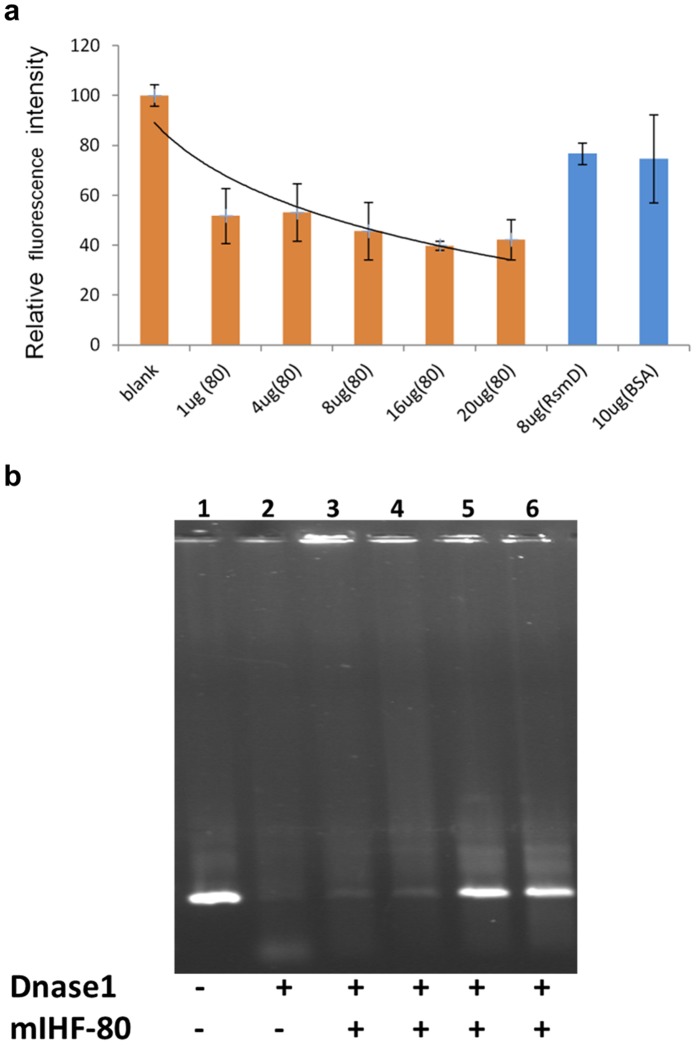
Role of mIHF-80 as a nucleoid associated protein. The role of mIHF-80 in DNA compaction was analyzed by (a) Ethidium bromide exclusion assay as well as (b) DNA protection assays. (a) Ethidium Bromide exclusion assay: A reaction mix containing plasmid DNA intercalated with Ethidium Bromide was incubated with 0 (no protein), 1, 4, 8, 16, or 20 µg mIHF-80 as indicated. The curve was normalized by taking the initial fluorescence reading (no protein) as 100%. The EtBr fluorescence decreases upto 40% upon DNA compaction with mIHF-80. A guideline indicating the decrease in fluorescence is also plotted. Rv2966c (8 µg) that binds DNA non-specifically [47] and BSA (10 µg) are used as controls, and show only marginal decrease in fluorescence. (b) DNA protection assay: mIHF-80 protects DNA from DNase digestion as increasing amount of mIHF-80 (lane 3: 7 µg, lane4: 35 µg, lane5: 70 µg, lane 6: 105 µg) is incubated with DNA. Negative (no DNase I) and positive controls (no mIHF-80) for the DNase activity are indicated in lanes 1 and 2, respectively.

The sequence non-specific nature of DNA binding by mIHF-80 suggested potential structural or architectural roles for this protein. A possible condensation of DNA into more compact structures was tested by Ethidium Bromide exclusion assay. Intercalation of EtBr into free DNA increases the quantum fluorescence yield of the dye, whereas there is a decrease in fluorescence intensity as the intercalated EtBr is excluded from DNA due to compaction and complex formation between mIHF-80 and DNA (Fig. 4a). Rv2966c that binds DNA non-specifically [49] and BSA (no known DNA binding role) were used as controls, and show only marginal decrease in fluorescence, confirming the decrease in fluorescence is due to DNA compaction and not merely DNA binding. The compaction of DNA by mIHF-80 and its role as a nucleoid-associated protein was further confirmed by the protection of DNA against degradation by DNase I upon binding of mIHF-80 (Fig. 4b).

### mIHF-80 Compacts DNA by a DNA Bending Mechanism

To elucidate the mechanism by which mIHF-80 binds and compacts DNA, direct imaging of complexes between mIHF-80 and DNA by Atomic Force Microscopy was carried out. As mIHF-80 binds both circular and linear DNA (Figure 3), the effects of binding of mIHF-80 to both circular and linear DNA molecules were studied using this approach. In order to ensure reliable conformations of mIHF-80 and DNA complexes are analyzed, DNA was allowed to equilibrate on the mica deposition surface and DNA lengths were verified to correspond to the expected length of linear and circular plasmid DNA molecules used in this study. Tables 1 and 2 describe the geometrical properties of the imaged circular and linear DNA. In addition, deposition of naked plasmid DNA examined in MilliQ water or in buffer used in protein-DNA complexes, (i.e. buffer condition equivalent to 150 ng protein-DNA complex; 150 mM NaCl, 5% Glycerol and 25 mM Tris-HCl, pH 8.0) was found to be similar (data not shown), and helped ensure that any changes in depositions is due to the properties of the protein-DNA complex and not buffer artefacts.

**Table 1:** Geometrical parameters of circular DNA+ mIHF-80 complexes.

	Plasmid control (per 10 bp): mIHF-80 (0)	Plasmid (per 10 bp):mIHF-80 (1∶1)	Plasmid (per 10 bp): mIHF-80 (1∶2)	Plasmid (per 10 bp):mIHF-80 (1∶4)	Plasmid (per 10 bp):mIHF-80 (1∶8)
Length^a^ (nm)σ^b^N	391.6361.0280	632.71114.5880	804.75185.1580	1690.95196.4480	901.1899.4280
Width^a^ (nm)σN	35.098.8680	19.123.7980	18.662.4180	18.052.3980	25.879.880
Height^a^ (nm)σN	1.410.1280	0.960.3580	1.020.1580	0.90.1380	1.370.0780
Angle^a^ (°)σN	133.023.450	98.618.650	79.813.950	79.113.350	54.216.250

a Indicated values are averages of molecules analyzed (N).

b σ is the Standard deviation for each parameter calculated over molecules analyzed (N).

**Table 2:** Geometrical parameters of linear DNA+ mIHF-80 complexes.

	Linear Plasmid DNA (per 10 bp): mIHF-80 (0)	Linear Plasmid DNA (per 10 bp):mIHF-80 (1∶2)	Linear Plasmid DNA (per 10 bp):mIHF-80 (1∶4)	Linear Plasmid DNA (per 10 bp):mIHF-80 (1∶8)
Length^a^ (nm)σ^b^N	1606.09133.4580	1117.45198.0680	839.73165.1380	873.16148.6480
Width^a^ (nm)σN	17.301.6380	17.451.7180	17.671.7680	17.671.8380
Height^a^ (nm)σN	0.840.2680	0.880.3480	1.080.2780	1.10.2080
Angle^a^ (°)σN	116.018.635	79.718.435	71.712.135	58.08.835

a Indicated values are averages of molecules analyzed (N).

b σ is the Standard deviation for each parameter calculated over molecules analyzed (N).

**Figure 5:**
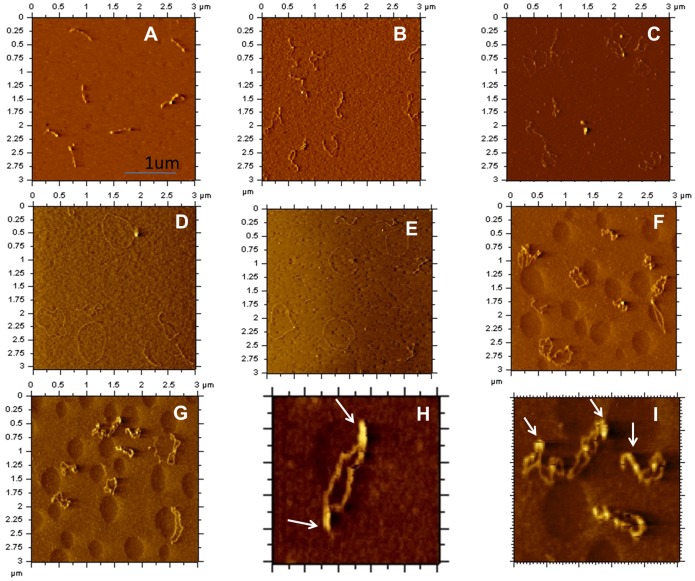
Representative AFM images of protein-DNA complexes formed between mIHF-80 and negatively supercoiled plasmid DNA. (A) Plasmid DNA in the absence of mIHF-80. (B)–(I): mIHF-80-pPROEX-HTc complexes with increasing mIHF-80 dimers per 10 base pair of DNA as follows; (B) 1, (C) 2, (D&E) 4, (F & G) 8. The scale of all images in A to G is (3 µm×3 µm). (H) & (I): Rigid nucleoprotein filaments identified in (F) and (G) above are shown at a slightly magnified scale of (1 µm×1 µm). The protein complexes are marked by arrows.

We first examined the effects of mIHF-80 binding to pPROEX-HTc, a 4779-base paired negatively supercoiled plasmid DNA. AFM images in absence of mIHF-80 showed uniform structures for the negatively supercoiled plasmid DNA (Fig. 5A). Fig. 5B–G show representative images of mIHF-80 and pPROEX-HTc complexes at an increasing ratio of mIHF-80 dimer to DNA base pairs (as indicated in the figure). The binding of mIHF-80 has an interesting effect on DNA, with initial opening of DNA at the ends (Fig. 5B), a mixed population of fully and partially open DNA circles (Fig. 5C), until completely relaxed DNA circles can be seen (Fig. 5D–E). Further increase in amounts of mIHF-80 results in several DNA bends in the DNA molecules leading to compaction of DNA (Fig. 5F–G). We were unable to obtain AFM images of the complexes at higher protein concentrations as mIHF-80 formed large aggregates on the mica under these conditions.

**Figure 6:**
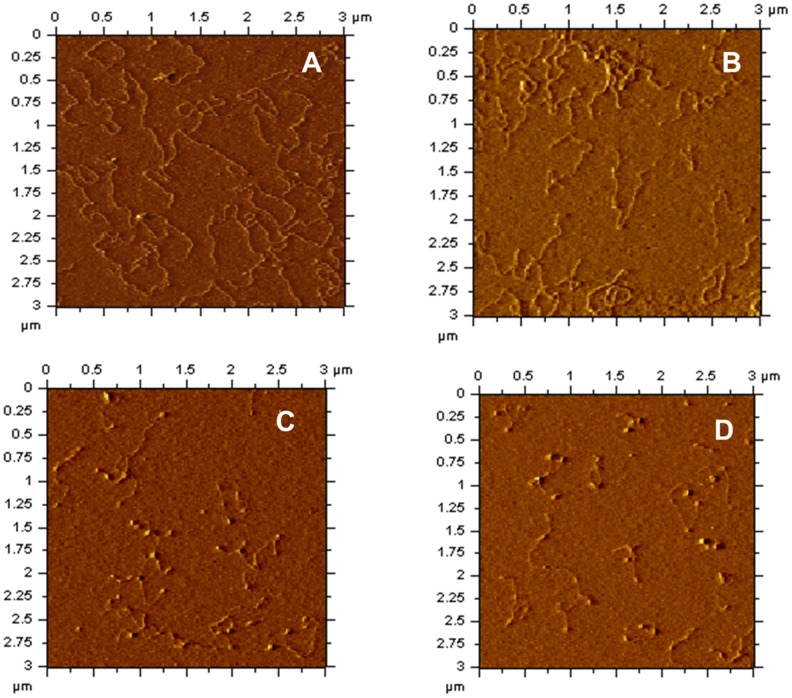
Representative AFM images of protein-DNA complexes formed between mIHF-80 and linear DNA. (A) Linear pPROEX-HTc in the absence of mIHF-80. (B)–(D): mIHF-80-pPROEX-HTc (linearized) complexes with following mIHF-80 dimer molecules per 10 base pairs of DNA; (B) 2, (C) 4 and (D) 8. The scale of all images is (3 µm×3 µm).

In order to gain insights into the mechanism of DNA compaction, AFM analysis was next carried out on linear DNA molecules. AFM images of linear DNA in the absence of protein shows uniform structures of even lengths (Fig. 6A). Further increase in protein concentrations results in increased compaction of the linear DNA as increasing protein to DNA ratios are used (Fig. 6B–D). These compactions appear to be a result of DNA bending introduced at multiple places in the DNA. The DNA bending introduced in linear DNA by mIHF-80 appears to be in the range of 60° to 80° (Table 2), while the average bending angle introduced by mIHF-80 at the highest ratio of 8∶1, is 58+/−8.8°.

The AFM analysis on linear and circular DNA indicates that mIHF-80 compacts DNA by a bending mechanism, suggesting that mIHF-80 is functionally and mechanistically similar to IHF of *E. coli*, despite lack of any sequence similarities. Moreover, AFM images with circular plasmid DNA indicates a peculiar pattern of DNA compaction with an initial opening up of the negatively supercoiled plasmid at low protein concentrations but further compaction at higher concentrations (Fig. 5). The dual compaction role of mIHF may be linked to a stoichiometry-dependent dual regulatory role for mIHF, in sync with antagonistic functions of other NAPs *in vivo*.

## Discussion

The present study shows that despite lack of sequence homology, mIHF participates in DNA compaction by a DNA bending mechanism similar to *E. coli* IHF. DNA bending and compaction by *E. coli* IHF is affected by protein, KCl and magnesium concentrations under non-specific DNA binding modes [50]. Changes in protein and salt concentrations induces different states of DNA bending and changes in DNA conformations. The DNA conformations thus induced may affect DNA compaction in early stationary phase when physiological IHF concentrations are very high [50]. IHF of other eubacteria is also expressed during late logarithmic and early stationary phase [51] and regulate the expression of virulence genes both in *Salmonella typhimurium* and *Vibrio cholera* [11], [21]. Expression of mIHF is also growth phase dependent and shows maximal expression during late logarithmic stages in *M. smegmatis* and *M. bovis* [28]. This suggests that like the *E. coli* IHF family, the temporal expression pattern of mIHF may be correlated with regulatory roles in late logarithmic stage of bacterial growth. Interestingly, mIHF homologue, SCO1480, affects antibiotic production during stationary phase of *Streptomyces coelicolor* [31], [32]. However, the non-specific nature of DNA binding as well as the role of mIHF in viability of *M. smegmatis* [28] and *M. tuberculosis* [29] suggests mIHF is likely to have an important role in regulation of not only specific pathways but as a general transcriptional regulator.

AFM imaging on circular, negatively supercoiled DNA with mIHF-80 shows a unique feature (Fig. 5). Although DNA bends are visible under all the investigated protein to DNA ratios with linear DNA (Fig. 6); low protein to DNA ratios opens up the DNA until fully relaxed circular DNA molecules are formed. This feature appears to be analogous to *E. coli* HU, another protein involved in DNA compaction by DNA bending. At low concentrations, *E. coli* HU binds DNA until rigid, completely open relaxed DNA circles are formed [52]. At high concentrations, HU dimers could be resolved on DNA and found to form rigid nucleoprotein filaments [53]. AFM studies with another protein, RdgC, involved in DNA recombination shows similar effects. At low RdgC to DNA ratios, there is unwinding of DNA and circular relaxed DNA is observed. At higher RdgC to DNA ratios, protein-protein interactions lead to DNA condensation [54]. The experimental limitation in our study did not allow us to investigate high protein to DNA ratios. At the highest investigated protein to DNA ratio of nearly one protein molecule (0.8 protein molecules) per base pair of DNA, larger complexes due to protein-protein interactions could be observed (Fig. 5H–I). A further increase in protein concentrations may lead to nucleoprotein filament formation analogous to *E. coli* HU. The ability of mIHF-80 to form stable oligomeric structures was also confirmed by cross-linking experiments (Fig. S6 in File S1). AFM studies with linear DNA confirm the mechanism of DNA compaction to be due to local bending of DNA molecules (Fig. 6). We hence propose a role for mIHF, wherein increasing protein to DNA ratios inside the cell result in formation of large protein complexes, leading to supercoiling as well as DNA bending and hence compaction. Interestingly, sIHF, the sequence homolog of mIHF-80 from *Streptomyces coelicolor* affects the topology of plasmid DNA and changes the DNA supercoiling states in presence of topoisomerase [52]. Although sIHF binds short DNA oligos, no mobility shift on gels is seen when sIHF alone is incubated with plasmid DNA [52]. mIHF, on the other hand, not only binds plasmid DNA efficiently (Fig. 3), AFM images suggest that it may affect the topology of DNA, when high protein:DNA ratios are used.

In another recent study, Lin et al [50] have shown that *E. coli* IHF induces different states of DNA bending, which may affect its physiological states including compaction. mIHF-80 also induces variable degree of bends in plasmid DNA that appears to be related to changes in protein concentration (Tables 1 and 2). Topological changes in plasmid DNA with high mIHF-80 concentrations appear to be a direct effect of these bends that increase the likelihood of protein-protein interactions in mIHF-80 resulting in large nucleoprotein complex formation. This feature of mIHF-80 may be utilized as a dynamic switch for mycobacterial growth in the cell. At low physiological levels during early growth to mid-logarithmic stages, mIHF may act as a protein activator (opening up of DNA, Fig. 5B–E), while with increasing mIHF concentrations at late logarithmic and stationary phase, mIHF may shift its role towards gene silencing by compacting DNA. A similar dual architectural role has been proposed for HU of *E. coli* where higher HU concentrations result in a rigid nucleoprotein filament [53]. However, the physiological significance of this hypothesis remains to be investigated.

The mycobacterial NAPs, appear to limited to Actinobacteria and other closely related bacteria and lack sequence homologs to *E. coli* counterparts of similar function. Mycobacterial NAPs namely, HU [47], [55], Lsr-2 [56] and H-NS [48] prefer A+T rich tracts for binding. These NAPs may have evolved this preference of DNA binding to protect its transcriptionally rich A+T tracts in the G+C rich mycobacterial genome. Alternately, some of its NAPs may have evolved to bind to G+C rich regions in the mycobacterial genome. DNA preference for mIHF and GroEL1 has not been identified so far, further investigations may help better understand the unique properties of these mycobacterial NAPs. The role of mIHF as a NAP in the present work suggests a functional and mechanistic conservation among bacterial NAPs despite lack of sequence similarities. In conclusion, we have shown that mIHF-80 of *M. tuberculosis* is an architectural protein involved in DNA compaction through a DNA bending mechanism in a non-sequence specific manner. Changes in physiological concentration of IHF concentration may change the degree of DNA bending affecting topology of DNA and hence function. Molecular details into these events need to be investigated further to implicate this protein in its nucleoid associated role and potential gene regulatory functions.

## Supporting Information

File S1 (DOCX)

File S2 (XLSX)
