# Supplementary material for: Integration Host Factor of Mycobacterium tuberculosis, mIHF, Compacts DNA by a Bending Mechanism
Source: PLoS One. 2013 Jul 26;8(7):e69985. doi: 10.1371/journal.pone.0069985 (PMC3724605; doi:10.1371/journal.pone.0069985)
Supplement: File S1 — (DOCX) [file pone.0069985.s001.docx]

# Integration Host Factor of *Mycobacterium tuberculosis*, mIHF, compacts DNA by a bending mechanism

# Mishra et al. (2013)

# Supplementary data:

Supplementary Figure S1: BLASTP with mIHF for all proteins in the archaeal and bacterial genomes in the UCSC Archaeal Genome Browser was carried out. As seen in the Figure, no hits with any other bacterial phylogenic group other than Actinobacteria were obtained, indicating that mIHF is unique to Actinobacteria.


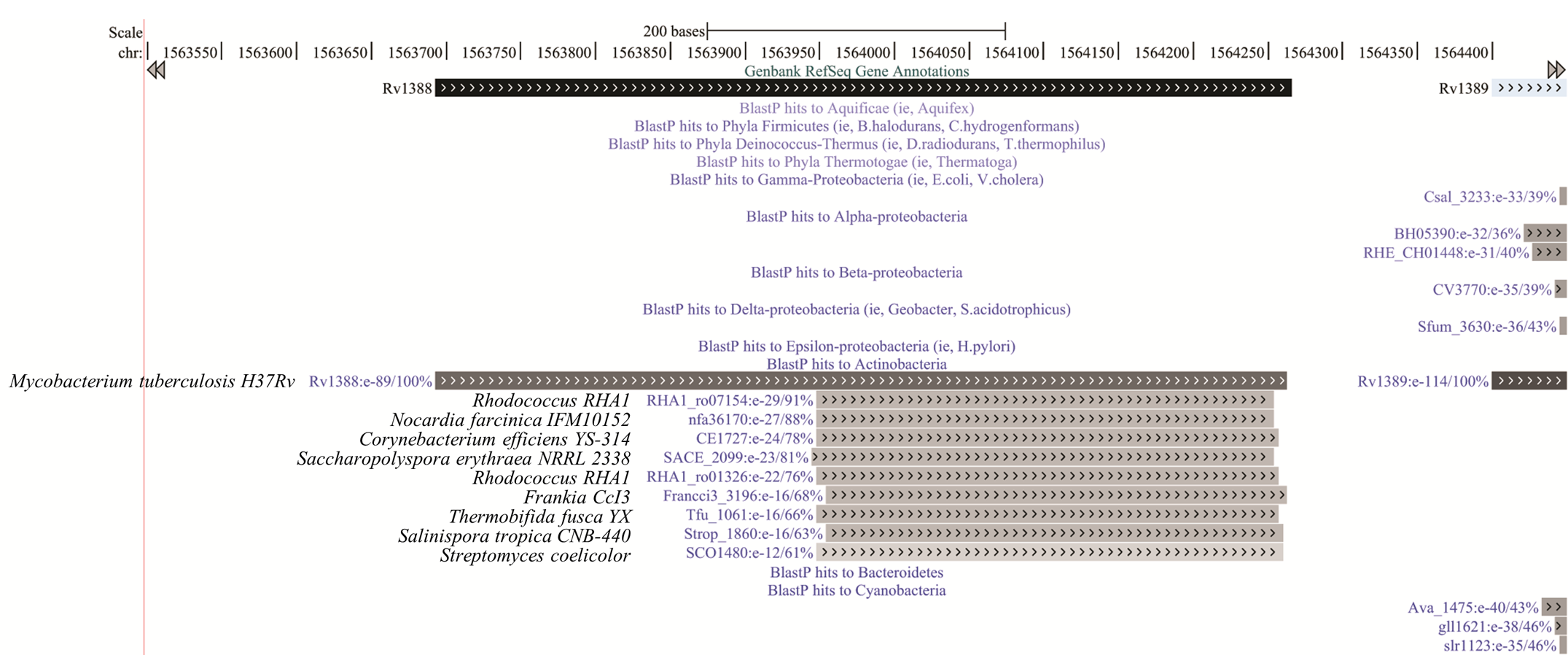


Supplementary Figure S2: Sequence alignment of representative mycobacterial IHF sequences. A multiple sequence alignment of representative mycobacterial IHF was carried out using CLUSTALW. As indicated, Rv1388 (*M. tuberculosis*) homologs are annotated to be 190, 111 or 105 residue long for different species. In the current study, we have cloned and purified the longer 111-residue protein for our work. The mycobacterial strains whose mIHF were used for multiple sequence alignment are mentioned next to the respective sequence.

CLUSTAL 2.1 multiple sequence alignment

M_tuberculosisH37Rv LLGNTIHVPCQPCRHGHGAPSRGLRGRPADRWPVARATPTLHVCPQNQGV 50

M_bovis_BCG MLGNTIHVPCQPCRHGHGAPSRGLRGRPADRWPVARATPTLHVCPQNQGV 50

M_tuberculosis_F11 MLGNTIHVPCQPCRHGHGAPSRGLRGRPADRWPVARATPTLHVCPQNQGV 50

M_tuberculosisH37Ra MLGNTIHVPCQPCRHGHGAPSRGLRGRPADRWPVARATPTLHVCPQNQGV 50

M_tuberculosis_KZN MLGNTIHVPCQPCRHGHGAPSRGLRGRPADRWPVARATPTLHVCPQNQGV 50

M_tuberculosis_CDC1551 --------------------------------------------------

M_marinum_M --------------------------------------------------

M_tuberculosis_str.Beijing --------------------------------------------------

M_tuberculosis_str._Erdman --------------------------------------------------

M_tuberculosis_str._Haarlem --------------------------------------------------

M_intracellulare_MOTT-02 --------------------------------------------------

M_leprae_TN --------------------------------------------------

M_smegmatis_str._MC2 --------------------------------------------------

M_ulcerans_Agy99 --------------------------------------------------

M_tuberculosisH37Rv GLDFVRKPEYGRLRWPAYPAGTNNDRLISMRDGGIVALPQLTDEQRAAAL 100

M_bovis_BCG GLDFVRKPEYGRLRWPAYPAGTNNDRLISMRDGGIVALPQLTDEQRAAAL 100

M_tuberculosis_F11 GLDFVRKPEYGRLRWPAYPAGTNNDRLISMRDGGIVALPQLTDEQRAAAL 100

M_tuberculosisH37Ra GLDFVRKPEYGRLRWPAYPAGTNNDRLISMRDGGIVALPQLTDEQRAAAL 100

M_tuberculosis_KZN GLDFVRKPEYGRLRWPAYPAGTNNDRLISMRDGGIVALPQLTDEQRAAAL 100

M_tuberculosis_CDC1551 -----------------------------MRDGGIVALPQLTDEQRAAAL 21

M_marinum_M -----------------------------MRDGGIVALPQLTDEQRAAAL 21

M_tuberculosis_str.Beijing -----------------------------------MALPQLTDEQRAAAL 15

M_tuberculosis_str._Erdman -----------------------------------MALPQLTDEQRAAAL 15

M_tuberculosis_str._Haarlem -----------------------------------MALPQLTDEQRAAAL 15

M_intracellulare_MOTT-02 -----------------------------------MALPQLTDEQRAAAL 15

M_leprae_TN -----------------------------------MALPQLTDEQRAAAL 15

M_smegmatis_str._MC2 -----------------------------------MALPQLTDEQRAAAL 15

M_ulcerans_Agy99 -----------------------------------MALPQLTDEQRAAAL 15

:**************

M_tuberculosisH37Rv EKAAAARRARAELKDRLKRGGTNLTQVLKDAESDEVLGKMKVSALLEALP 150

M_bovis_BCG EKAAAARRARAELKDRLKRGGTNLTQVLKDAESDEVLGKMKVSALLEALP 150

M_tuberculosis_F11 EKAAAARRARAELKDRLKRGGTNLTQVLKDAESDEVLGKMKVSALLEALP 150

M_tuberculosisH37Ra EKAAAARRARAELKDRLKRGGTNLTQVLKDAESDEVLGKMKVSALLEALP 150

M_tuberculosis_KZN EKAAAARRARAELKDRLKRGGTNLTQVLKDAESDEVLGKMKVSALLEALP 150

M_tuberculosis_CDC1551 EKAAAARRARAELKDRLKRGGTNLTQVLKDAESDEVLGKMKVSALLEALP 71

M_marinum_M EKAAAARRARAELKDRLKRGGTNLTQVLKDAETDEVLGKMKVSALLEALP 71

M_tuberculosis_str.Beijing EKAAAARRARAELKDRLKRGGTNLTQVLKDAESDEVLGKMKVSALLEALP 65

M_tuberculosis_str._Erdman EKAAAARRARAELKDRLKRGGTNLTQVLKDAESDEVLGKMKVSALLEALP 65

M_tuberculosis_str._Haarlem EKAAAARRARAELKDRLKRGGTNLTQVLKDAESDEVLGKMKVSALLEALP 65

M_intracellulare_MOTT-02 EKAAAARRARAELKDRLKRGGTNLTQVLKDAETDEVLGKMKVSALLEALP 65

M_leprae_TN EKAAAARRARAELKDRLKRGGTNLTQVLKDAESDEVLGKMKVSALLEALP 65

M_smegmatis_str._MC2 EKAAAARRARAELKDRLKRGGTNLKQVLTDAETDEVLGKMKVSALLEALP 65

M_ulcerans_Agy99 EKAAAARRSRAELKDRLKRGGTNLTQVLKDAETDEVLGKMKVSALLEALP 65

********:***************.***.***:*****************

M_tuberculosisH37Rv KVGKVKAQEIMTELEIAPTRRLRGLGDRQRKALLEKFGSA 190

M_bovis_BCG KVGKVKAQEIMTELEIAPTRRLRGLGDRQRKALLEKFGSA 190

M_tuberculosis_F11 KVGKVKAQEIMTELEIAPTRRLRGLGDRQRKALLEKFGSA 190

M_tuberculosisH37Ra KVGKVKAQEIMTELEIAPTRRLRGLGDRQRKALLEKFGSA 190

M_tuberculosis_KZN KVGKVKAQEIMTELEIAPTRRLRGLGDRQRKALLEKFGSA 190

M_tuberculosis_CDC1551 KVGKVKAQEIMTELEIAPTRRLRGLGDRQRKALLEKFGSA 111

M_marinum_M KVGKVKAQEIMTELEIAPTRRLRGLGDRQRKALLEKFGSA 111

M_tuberculosis_str.Beijing KVGKVKAQEIMTELEIAPTRRLRGLGDRQRKALLEKFGSA 105

M_tuberculosis_str._Erdman KVGKVKAQEIMTELEIAPTRRLRGLGDRQRKALLEKFGSA 105

M_tuberculosis_str._Haarlem KVGKVKAQEIMTELEIAPTRRLRGLGDRQRKALLEKFGSA 105

M_intracellulare_MOTT-02 KVGKVKAQEIMTELEIAPTRRLRGLGDRQRKALLEKFGS- 104

M_leprae_TN KVGKVKAQEIMTELDIAPTRRLRGLGERQRKALLEKFGSA 105

M_smegmatis_str._MC2 KVGKVKAQEIMTELEIAPTRRLRGLGDRQRKALLEKFDQS 105

M_ulcerans_Agy99 KVGKVKAQEIMTELEIAPTRRLRGLGDRQRKALLEKFGSA 105

**************:***********:**********..

Supplementary Figure S3**:** View of genomic locus of Rv1388 with gene predictions and peptide identifications according to (a) Wolfe et al, 2010 and (b) de Souza et al, 2011: Genome annotation of Rv1388 in Genbank (top bar) versus Gene predictions by the indicated algorithms (GeneMark, Prodigal and Glimmer) were visualized in UCSC genome browser. The peptides from the respective proteome analysis are indicated below.

**
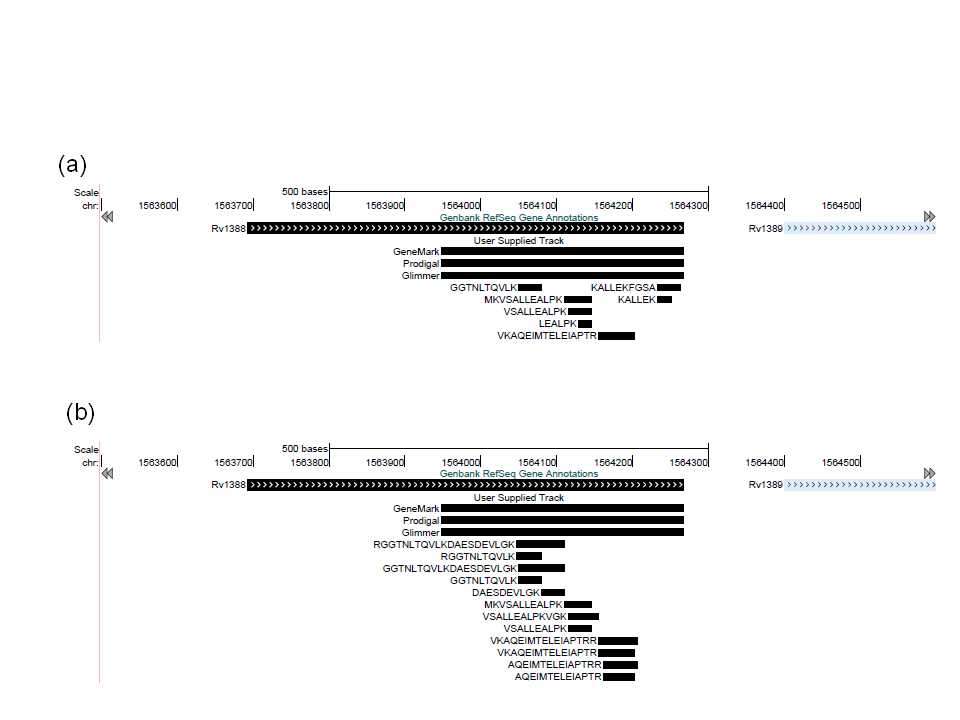
**

Supplementary Figure S4: Analysis of aliquots (6μg each) of (a) Ulp1 treated His_10_-Smt3-mIHF-80 fusion (lane 1) and (b) purified mIHF-80 by SDS−PAGE. The positions of the separated Smt3 tag (A) and mIHF-80 (B) are indicated. The Coomassie blue-stained gel is shown. Position and sizes of molecular weight markers (in kDa) are indicated in *lane M* in both panels.


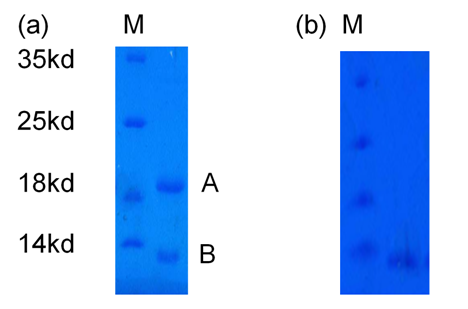


Supplementary Figure S5: Gel filtration analysis of oligomeric state of mIHF-80. mIHF-80 was analyzed for its oligomeric state by gel permeation chromatography over Superdex-75 column (GE Life Sciences). 300 µg of mIHF-80 was loaded onto Superdex-75 and eluted with buffer C described in the Methods section, containing 50mM Tris-HCl buffer, pH 8.0, 300mM NaCl and10 % (v/v) glycerol. A single peak suggesting a homodimeric form of mIHF-80 of approximate molecular weight of 24 kDa was observed.


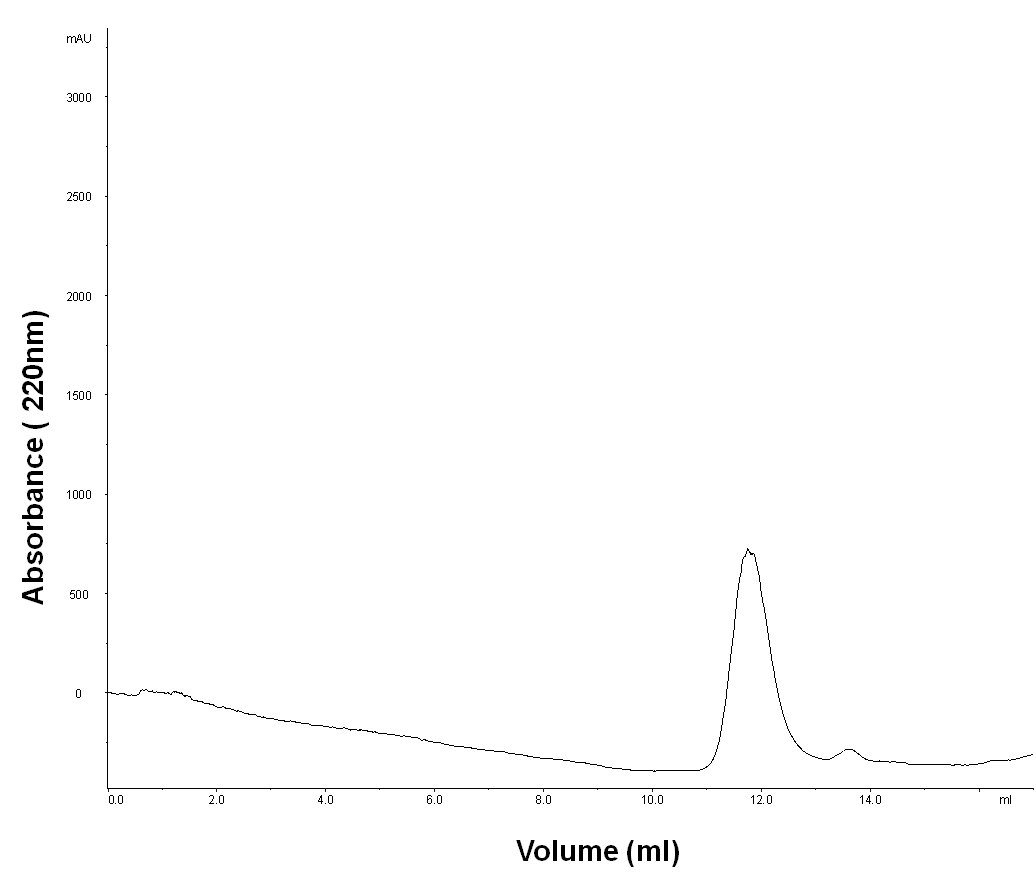


Supplementary Figure S6: To analyze the oligomeric states of mIHF-80 in solution, the protein was crosslinked with different concentrations of gluteraldehyde (2.5%, 1.5%, 1.0% and 0.5%) (lanes 2-5). The reaction was carried out in HBS-EP buffer (GE Life Sciences): 0.01 M HEPES pH 7.4, 0.15 M NaCl, 0.005% v/v Surfactant P20 with indicated amounts of gluteraldehyde and stopped by adding 1M Tris-HCl, pH 8.0. The samples were loaded onto a 15% polyacrylamide gel and the oligomeric state was calculated by comparing with standard molecular weight protein marker (lane M) (Fermentas). Purified recombinant mIHF-80 without cross-linker is shown in lane 1. The dimeric, trimeric and tetrameric states of mIHF-80 are indicated by arrows.


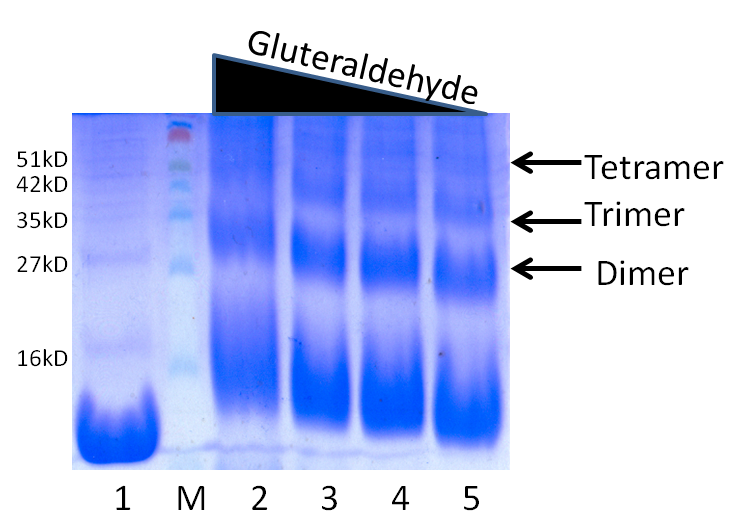


Supplementary Table S**1**: Details of the peptides corresponding to Rv1388, as identified from proteome profile of *M. tuberculosis* H37Rv (Kelkar et al, 2011) are given in attached Excel sheet (File S2).
